# Decoding Leukemic Stem Cells in AML: From Identification to Targeted Eradication

**DOI:** 10.3390/diseases14020050

**Published:** 2026-01-30

**Authors:** Elisavet Apostolidou, Vasileios Georgoulis, Dimitrios Leonardos, Leonidas Benetatos, Eleni Kapsali, Eleftheria Hatzimichael

**Affiliations:** 1 Department of Haematology, University Hospital of Ioannina, 45 500 Ioannina, Greece; e.apostolidou@uoi.gr (E.A.); v.georgoulis@uoi.gr (V.G.); d.leonardos@uoi.gr (D.L.); lbenet@uoi.gr (L.B.); ekapsali@uoi.gr (E.K.); 2 Department of Haematology, Faculty of Medicine, School of Health Sciences, University of Ioannina, 45 500 Ioannina, Greece

**Keywords:** acute myeloid leukemia, leukemic stem cells, immunophenotype, targeted treatment

## Abstract

Acute myeloid leukemia (AML) continues to pose significant therapeutic challenges, with high relapse rates driven largely by leukemic stem cells (LSCs), a rare, therapy-resistant population with self-renewal capacity, niche adaptation, and the ability to re-initiate disease. In this state-of-the-art review, we synthesize recent advances in LSC biology, addressing (i) how LSCs differ functionally and phenotypically from normal hematopoietic stem cells (HSCs), (ii) practical approaches for LSC quantification using multiparameter flow cytometry and LSC-enriched marker panels, (iii) the dysregulated signaling, metabolic and epigenetic programs that enable LSC persistence under chemotherapy and contribute to measurable residual disease, and (iv) current therapeutic strategies targeting LSC eradication, including antibody-based therapies, apoptosis and metabolic inhibitors, and emerging epigenetic agents. We also examine the key translational barriers, particularly antigen overlap with normal progenitors, microenvironmental protection, and the need for assay harmonization, while proposing a practical framework for integrating LSC assessment into risk stratification and therapeutic development.

## 1. Introduction

Acute myeloid leukemia (AML) represents a highly heterogeneous group of hematologic malignancies driven by diverse cytogenetic aberrations and molecular mutations. This heterogeneity arises from the combinatorial diversity of driver mutations, the variable cell of origin along the hematopoietic hierarchy, and the influence of patient-specific factors including germline variants and microenvironmental interactions. The disease arises from the clonal expansion of hematopoietic precursors that have acquired a differentiation blockade and uncontrolled proliferative capacity. This results in the accumulation of undifferentiated blast cells within the bone marrow microenvironment (BMM) and peripheral blood, which progressively disrupts normal hematopoiesis and leads to bone marrow failure [1,2].

In the era of next-generation sequencing (NGS), numerous somatic mutations have been identified in hematopoietic stem cells (HSCs) and progenitor cells, influencing disease prognosis and modifying the current risk stratification according to the European Leukemia Net (ELN) 2022 [2]. Clinical outcome and prognosis are significantly influenced by underlying molecular and cytogenetic abnormalities along with age at diagnosis and comorbidities. Despite this expansion in the mutational landscape of AML, the therapeutic combination of cytarabine and anthracycline, “3 + 7”, as an induction treatment remains the standard of care for newly diagnosed and fit patients [2,3]. However, relapsed or refractory AML is a common scenario and the most challenging aspect, while long-term overall survival (OS) rates are only 40–50% in younger patients and even worse in older adults, with median OS being typically under one year [3].

Leukemic stem cells (LSCs), also referred to as leukemia-initiating cells (LICs), are now established as a distinct cell subpopulation in BM, acquiring distinctive properties of quiescence, self-renewal, and proliferation through clonal outgrowth and continuous genetic and epigenetic modifications. Several studies revealed that the existence of a small subpopulation of LSCs in the BM niche is associated with chemotherapy resistance and thus disease relapse as they can repopulate leukemia cells. The BMM plays a crucial role not only in supporting hematopoiesis through myelopoiesis and lymphopoiesis but also in leukemogenesis, disrupting normal hematopoietic stem cell niches and creating a permissive microenvironment for LSC survival and proliferation [1,4,5].

In this state-of-the-art narrative review, we prioritize clinically actionable evidence and recent translational studies to provide (i) a translational framework for LSC identification across AML subtypes, (ii) a practical integration of LSC measurement with measurable residual disease (MRD), and (iii) a 2025-updated appraisal of LSC-directed therapies using a targetability lens that prioritizes clinical feasibility, safety, and resistance biology.

## 2. Biological Features of LSCs

The first in vivo experiments that demonstrated the existence and leukemogenic potential of LSCs were those carried out by Lapidot, Dick, and colleagues. They showed that primitive CD34^+^/CD38^−^ cells from patients with AML could engraft in mice with severe common immunodeficiency (SCID), self-renew, proliferate, and differentiate into leukemic blasts, generating leukemia in mice, and were considered as LICs [6,7].

Following these discoveries, several efforts have been made to establish a theory that best explains the leukemia-initiating process. Of these, the most widely accepted seems to be the hierarchical model which resembles the organization of normal haematopoiesis. This consideration was based on evidence from functional studies indicating that LSCs share biological similarities with normal HSC and, simultaneously, can sustain long-term leukemogenesis [7]. In the continuous, physiological process of hematopoiesis, a rare population of multipotent HSCs differentiate into progenitor cells committed to specific blood cell lineages, while retaining self-renewal capacity, thereby preserving the HSC pool. This organization is recapitulated in AML, with rare LSCs standing at the hierarchical apex and giving rise to more committed cells, the leukemic blasts, thus sustaining the whole leukemic cell mass [8,9,10].

However, the cell of origin in AML remains a topic of ongoing debate. According to the most widely accepted theory, at some timepoint, a first mutation of an HSC may affect epigenetic genes or genes of histone modification such as *DNMT3A* or *ASXL1*, but without the ability to generate leukemia immediately. After a substantial period, another mutation (second hit) in genes regulating cell signaling or cellular proliferation may convert the pre-leukemic HSCs into actual LSCs, which can, in turn, initiate leukemia [8,9].This multi-step process is later discussed in detail. However, Cozzio et al. showed that the *MLL* fusion gene, recurrently found in AML, can provide HSCs and progenitor cells with immortality and self-renewal capacity, converting them into LICs and, hence, AML could actually arise not only from stem cells at the top of the hierarchy, but also from more mature committed myeloid progenitors [11].

As previously mentioned, LSCs have several biological characteristics, some of which are similar to those of normal HSCs, underlying their stemness and close relation to their normal counterparts. Like normal HSCs, LSCs comprise a very rare cellular population in the BM compartment, approximately 0.1%, compared to the expanded leukemic blast population, and are mainly in a quiescent state [12]. Additionally, LSCs have self-renewal capacity, which means that they can replicate and preserve their initial population. At the same time, LSCs can differentiate into more mature cellular stages, mainly leukemic blasts, which gradually lose their self-renewal and differentiation capacity. Furthermore, stem cells are characterized by drug resistance, which, in the case of HSCs, is responsible for normal hematopoiesis restoration after chemotherapy, but it could also be the reason for AML relapse after achieving complete remission (CR), when it comes to LSCs [8]. On the other hand, LSCs have acquired genetic mutations, as previously discussed, and phenotypic characteristics such as altered metabolic properties or aberrant RNA editing, which confer them survival and proliferative advantages translated into leukemogenic potential and, therefore, differentiate them from normal HSCs. Such inherent differences have been exploited by scientists to develop therapeutic strategies targeting LSCs while sparing normal HSCs. For example, in AML, LSCs are characterized by low levels of reactive oxygen species (ROS) and are highly dependent on mitochondrial catabolism of amino-acids and fatty acids and oxidative phosphorylation (OXPHOS) rather than glycolysis which represent the main carbohydrates metabolic pathway in non-malignant HSCs or bulk leukemic blast cells [13,14]. With this rationale, several molecules targeting different pathways of carbohydrates metabolism are under assessment. Some of them (e.g., tigecycline) aim at mitochondrial translation of electron transport chain enzymes involved in OXPHOS of LSCs, while others such as 2-deoxyglucose (2-DG), TT-232 and other glycolytic enzymes inhibitors mainly interrupt the glycolytic process of leukemic blasts [15,16]. When it comes to lipids, leukemic blasts’ altered lipid metabolism is characterized by accumulation of intracellular lipid droplets, which act not only as a cytoplasmic lipid storage to sustain the malignant cell’s high metabolic needs and membrane synthesis, but also regulate energy production and can contribute to chemoresistance through drug sequestration and inactivation. Thus, targeting lipid droplets biogenesis with several agents such as PPARγ ligands, etomoxir and chenodeoxycholic acid (CDCA) has been investigated in AML cellular models as a potential therapeutic intervention [17,18]. Moreover, it has been found that with alternative splicing, LSCs can enhance the expression of anti-apoptotic molecules, including BCL2 and MCL1, or molecules that enable immune evasion, such as CD47 [12]. Finally, LSCs have developed special mechanisms of crosstalk with the BMM that promote their survival, proliferation, and immune evasion, as further explained in the next section.

The biological properties of LSCs are governed by several molecular signaling pathways, the targeting of which could have therapeutic implications. Some of these multistep key-signaling pathways are aberrantly exploited by LSCs to promote their survival and clonal expansion, gaining an advantage over non-malignant hematopoietic cells in the BM. For instance, the NOTCH signaling pathway, important for tissue development, is active in normal hematopoiesis, but the overexpression of the Notch intracellular domain (NICD) can immortalize stem cells in the case of AML [19]. Another highly conserved signaling pathway consists of the binding of Wnt with its receptor, which causes the accumulation of β-catenin in the nucleus, where it promotes the expression of genes involved in the proliferation of cells, HSCs included [10]. Overexpression of β-catenin has been documented in LSCs, pointing to a possible key role of this pathway in LSC expansion [20]. The JAK/STAT pathway is also integral to LSC biology, as STAT5B has been shown to activate genes associated with quiescence and self-renewal [21]. Data have been rather conflicting regarding the role of the Hedgehog pathway in the pathogenesis of AML, since some studies found that this signaling pathway is not necessary for the maintenance of LSCs, while others support an essential role of the pathway since overexpression of *GLI1*, a key-mediator of the Hedgehog pathway, has been associated with worse outcomes and increased chemo-resistance [10,22,23,24]. Other signaling pathways seem to be of greater importance in specific AML subtypes. For instance, LSCs in t(8;21) AML seem to aberrantly activate the VEGF/IL-5 pathway, which allows them to re-enter the cell cycle and preserve their self-renewal ability, while FoxM1 regulates cellular proliferation specifically in LSCs of *MLL*-rearranged AML [25]. Ongoing research reveals many more signaling pathways involved in biological processes of LSCs, including BMI-1, PI3K-AKT, AHR and PDK1, which could provide multiple promising targets for therapeutic interventions [23]. Experimentally, the study of LSC biology relies on specific cell line models. Cell lines such as KG-1 and KG-1a are enriched for CD34^+^CD38^−^ stem-like populations and are commonly used to model LSC behavior, whereas lines such as HL-60, U937, and THP-1 represent more differentiated blast populations. Primary patient-derived xenograft (PDX) models remain the gold standard for functional LSC assessment, as they preserve the hierarchical organization and microenvironmental dependencies observed in patients.

## 3. The Role of LSCs and the BMM in Leukemogenesis

In recent years, neoplastic stem cells have been categorized into two distinct types, premalignant (pre-leukemic) neoplastic stem cells (pre-L-NSCs) and leukemic (malignant) neoplastic stem cells. Myeloid leukemogenesis is driven by the dysregulation of numerous molecular mechanisms, including aberrant epigenetic modifications, transcription factor activity, and signal transduction pathways. Furthermore, defects in DNA damage response pathways and cell cycle checkpoints promote genomic instability, while the BMM contributes by providing essential survival signals and niche protection. Ultimately, this complex evolution involves the acquisition of somatic mutations, clonal selection, dynamic interactions with the BM niche, and immune evasion [26].

### 3.1. LSCs and Leukemogenesis

AML evolves through a multistep process in which sequential mutations transform normal HSCs into pre-leukemic clones and, eventually, leukemia-initiating LSCs. Pre-LSCs typically generate small clonal populations and retain many functional properties of normal HSCs, whereas fully transformed LSCs possess the capacity to initiate and sustain overt leukemia [27]. Clonal hematopoiesis (CH) refers to the age-associated expansion of HSCs harboring somatic mutations in leukemia-associated genes and comprising a pre-leukemic state, constituting the clonal hematopoiesis of indeterminate potential (CHIP) [28]. Another significant issue is the term clonal hematopoiesis of oncogenic potential (CHOP), which marks the conversion of a non- or pre-malignant condition into an overt malignancy. The acquisition of one or more CHIP mutations is a prerequisite for the transformation of a normal stem cell into a pre-LSC, while LSCs are frequently characterized by the presence of two or more CHIP mutations or one CHOP mutation [27].

**Figure 1:**
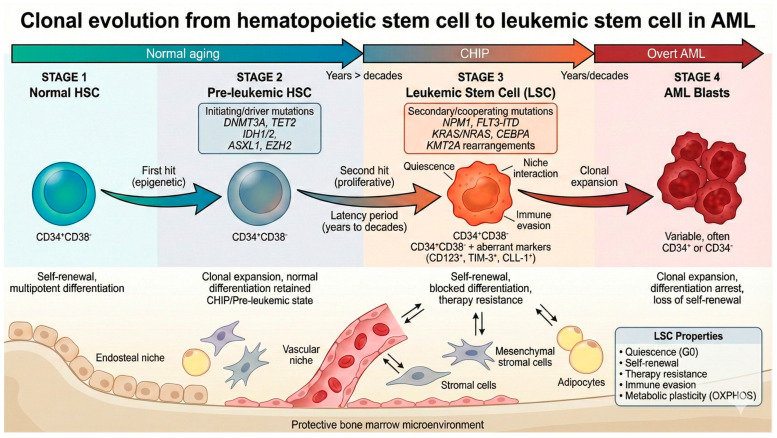
Multistep clonal evolution from normal HSC to LSC in AML. Normal HSCs acquire initiating mutations in epigenetic modifier genes (*DNMT3A*, *TET2*, *IDH1/2*, *ASXL1*), generating pre-leukemic HSCs that expand clonally but retain differentiation capacity (CHIP). After a latency period of years to decades, acquisition of secondary mutations in proliferation-associated genes (*NPM1*, *FLT3-ITD*, *KRAS*/*NRAS*, *KMT2A* rearrangements) transforms pre-leukemic HSCs into LSCs capable of initiating overt leukemia. LSCs reside within protective bone marrow niches and exhibit quiescence, self-renewal, therapy resistance, and immune evasion. LSCs give rise to rapidly proliferating leukemic blasts with blocked differentiation. Aberrant surface markers (CD123, TIM-3, CLL-1) distinguish LSCs from normal HSCs and represent potential therapeutic targets; illustration created with Gemini 3.

Initiating or “driver” mutations occur in epigenetic modifiers that regulate cytosine methylation, such as *DNMT3A* (DNA methyltransferase 3 alpha), *IDH 1/2* (isocitrate dehydrogenase 1/2), and *TET2* (tet methylcytosine dioxygenase 2), as well as in histone modifiers, such as *ASXL1* (additional sex combs-like) and *EZH2* (enhancer of zeste homolog 2). These early alterations confer a selective advantage and enhanced self-renewal capacity to HSCs and progenitor cells but are generally insufficient to induce overt leukemia. Subsequently, additional somatic mutations are necessary to promote uncontrolled proliferation and leukemic transformation, as preleukemic and late events, like *NPM1* (nucleophosmin 1), *FLT3-ITD* (internal tandem duplication of the gene *FLT3*), *KRAS*/*NRAS* (Kirsten rat sarcoma viral oncogene homolog/neuroblastoma rat sarcoma viral oncogene homolog) and *CEBPA* (CCAAT/enhancer-binding protein alpha) driving pre-LSCs into fully transformed LSCs (Figure 1) [1,22,29]. LSCs generate leukemic blasts possessing leukemia-associated mutations and exhibit a blockade in differentiation. In contrast, pre-LSCs retain multilineage differentiation potential despite carrying recurrent initiating mutations, giving rise to phenotypically normal progenitor cells that nonetheless harbor pre-leukemic genetic lesions and may persist during remission [1,28].

### 3.2. BM Niche and Leukemogenesis

The BM is a complex hematopoietic organ comprising a dynamic microenvironment of heterogeneous cellular populations, vascular and neural networks, and non-cellular components organized into functional niches [30]. The BM niche is the specialized microenvironment that supports HSCs and regulates their self-renewal, differentiation, and proliferation to maintain hematopoietic homeostasis [31]. HSCs reside in a specialized, physiologically hypoxic milieu where their quiescence and undifferentiated state are preserved through direct cell–cell contacts and soluble factor-mediated interactions within the BMM. Based on anatomical localization, BM niches are commonly categorized into the endosteal (osteoblastic) niche, located adjacent to the endosteum, and the vascular niche, associated with blood vessels and the surrounding perivascular matrix. The BMM includes stromal and immune populations (T and B lymphocytes, macrophages, natural killer cells, and dendritic cells), as well as osteoblasts, adipocytes, perivascular mesenchymal stem/stromal cells, endothelial cells, and neural elements [32,33]. Non-cellular components, including growth factors, cytokines, chemokines, and extracellular matrix constituents are also essential for controlling hematopoiesis and sustaining stem cell homeostasis. The endosteal niche has been associated with HSC maintenance and quiescence, whereas the vascular niche contributes to the trafficking and release of more mature hematopoietic precursors into the circulation [33].

In AML, a complex bidirectional interaction is established between LSCs and BMM, supporting both disease initiation and progression. LSCs actively remodel the healthy niche, creating a permissive microenvironment that promotes leukemic dominance, expansion, and protection from cytotoxic chemotherapy [34]. Reported AML-associated alterations of the BMM include (i) increased pro-angiogenic signaling and angiogenesis; (ii) lipolysis and reduction in BM adipose tissue; (iii) neuropathic changes involving the sympathetic nervous system; (iv) dysregulation of cytokine and chemokine networks, accompanied by upregulation of adhesion molecules that activate pro-survival signaling pathways; (v) adaptation to hypoxic conditions with suppression of endogenous reactive oxygen species (ROS) production; (vi) reprogramming of mesenchymal stem/stromal cells to support leukemic proliferation; and (vii) escape from immune surveillance, including through immune checkpoint dysregulation [30,35]. A deeper mechanistic understanding of this leukemia–niche interplay may enable the development of strategies that disrupt microenvironmental protection and overcome therapy resistance in AML [30,35].

## 4. Immunophenotypic Identification of LSCs

Multiparameter flow cytometry (MFC) enables the immunophenotypic identification of LSC-enriched populations in AML using fluorochrome-labeled antibody panels that target defined surface antigen patterns. Most clinical and translational data derive from CD34^−^positive AML, where LSCs are enriched within the CD34^+^CD38^−^ compartment. Because this gate overlaps with normal HSCs, the practical challenge lies not in enrichment alone but in discriminating leukemic CD34^+^CD38^−^ events from normal HSCs using aberrant or LSC-associated markers. A pragmatic approach involves first defining the CD34^+^CD38^−^ compartment and subsequently identifying leukemic events through LSC-associated markers and lineage aberrancies. Zeijlemaker et al. developed and validated a single 8-color tube for the efficient quantification of CD34^+^CD38^−^ LSCs at diagnosis and during follow-up. This panel combines backbone markers (CD45, CD34, CD38) with LSC-associated markers (CD45RA, CD123, CD33, CD44) and a cocktail of additional aberrancy markers, including CLL-1, TIM-3, CD7, CD11b, CD22, and CD56, within a single fluorescence channel. This design facilitates broad applicability while preserving analytical feasibility in routine laboratory workflows [36,37].

For more accurate identification and discrimination of LSCs from HSCs, additional markers frequently used across studies include CD123 (IL-3Rα), CD44, CD33, CLL-1 (CLEC12A/CD371), TIM-3 (CD366), CD45RA, CD96, IL1RAP, and CD200. The diagnostic utility of these markers stems from their aberrant overexpression or asynchronous presence on LSCs compared to the phenotype of normal HSCs, thereby enabling the specific isolation and tracking of the malignant reservoir. Crucially, distinguishing LSCs from normal HSCs becomes challenging in regenerating BM (e.g., post-chemotherapy) where normal HSCs expand. In this context, CD90 (Thy-1) serves as a vital negative selection marker, as regenerating HSCs are strictly CD90-positive, whereas LSCs in most AML cases are CD90-negative. LSC-enriched compartments may also exhibit aberrant expression of lineage-associated antigens such as CD7, CD11b, CD22, CD56, and occasionally CD19, which can be leveraged as “different-from-normal” signals to distinguish leukemic stem-like populations from normal HSCs. Table 1 summarizes key markers reported on LSCs and HSCs [38,39,40].

MRD refers to residual leukemic cells detectable below the morphological threshold of 5% blasts by conventional cytomorphology after initial therapy, reflecting the clearance of the leukemic blast population [49,53]. MRD positivity is a strong prognostic biomarker associated with increased relapse risk and inferior OS and disease-free survival (DFS), and is thus increasingly incorporated as an efficacy-response endpoint in therapeutic development [54]. Common MRD methodologies include MFC, polymerase chain reaction (PCR) assays targeting recurrent gene fusions or specific genomic alterations, and NGS, each offering distinct sensitivity and specificity profiles [55]. According to 2017 ELN recommendations, two core strategies underpin MFC-based MRD assessment: the leukemia-associated immunophenotype (LAIP) approach and the different-from-normal (DfN) approach. These frameworks improve monitoring accuracy and support standardized reporting, with MRD positivity commonly defined at a threshold of 10^−4^ or higher [49,53]. Incorporating LSC-directed MFC assays may further enhance sensitivity and predictive accuracy by focusing on therapy-resistant subpopulations enriched for relapse-initiating capacity. ELN recommendations also highlight the prognostic significance of LSC quantification, typically defined immunophenotypically as CD34^+^CD38^−^ cells co-expressing aberrant markers not usually present on normal HSCs, such as CD45RA (PTPRC), CLL-1 (CLEC12A), or CD123 (IL3RA) [53].

Within this framework, MRD quantifies the bulk residual leukemia burden, whereas LSC-enriched readouts provide complementary information on stemness-associated persistence and relapse biology. Combining MFC-based MRD with LSC-oriented phenotyping may therefore improve risk stratification, therapeutic monitoring, and relapse prediction beyond conventional MRD approaches alone [54,55]. In practice, an LSC-enriched MFC readout is best interpreted as an adjunct to MRD rather than a standalone substitute for established methodologies and is particularly valuable when it identifies a persistent CD34^+^CD38^−^ aberrant compartment despite apparent cytoreduction. Recent standardization efforts by the HOVON group emphasize the technical stringency required for LSC detection. Reuvekamp et al. demonstrated that assessing at least 1 million (and ideally up to 4 million) events is requisite for high-sensitivity LSC-MRD monitoring. Furthermore, they highlighted that combining LSC assessment with conventional MRD significantly refines risk stratification, with double-negative patients (MRD−/LSC−) achieving superior 3-year survival rates (80%) compared to double-positive cases (45%), thereby validating the clinical utility of the dual-assay approach [56].

## 5. The Contribution of LSCs in Relapse and AML Progression

The management of AML remains challenged by high rates of relapse. LSCs are fundamentally responsible for the re-initiation of leukemic events due to their intrinsic capacity for unlimited self-renewal and their ability to enter a protective quiescent state. Relapse occurs when residual leukemic cells, comprising a subclone of blasts and quiescent LSCs, either evade initial treatment or remain undetected at diagnosis. Due to the unique biological characteristics of LSCs, conventional chemotherapy, which mainly targets rapidly proliferating blasts, often fails to eliminate the dormant LSC population leading to MRD positivity. These surviving LSCs subsequently adapt by undergoing phenotypic changes and acquiring additional somatic mutations, contributing to disease recurrence. Consequently, developing more effective therapies to eliminate LSCs remains a critical unmet need [1].

The resistance mechanisms of LSCs are complex and involve multiple pathways. The majority of LSCs possess the capacity to enter a quiescent or dormant state (G0), allowing them to evade the cytotoxic effects of agents, such as cytarabine and anthracyclines, that primarily target rapidly dividing cells. The efflux pump activity, ATP-binding cassette (ABC) transporters (P-gp, BCRP, MRP1), is overexpressed in LSCs expelling chemotherapeutic agents and protecting them from the cytotoxic effects of chemotherapy. Furthermore, LSCs exploit the BMM, particularly by remodeling the endosteal niches to survive and protect themselves from the cytotoxic effects of chemotherapy. The upregulation of anti-apoptotic proteins (e.g., BCL-2, MCL-1) contributes significantly to apoptosis resistance and survival of LSCs. Chemotherapeutic drugs induce DNA damage, driving cells to premature senescence, but several studies revealed that LSCs demonstrate senescent resistance and DNA repair mechanisms compared to HSCs. Metabolically, LSCs exhibit metabolic flexibility, depending more on OXPHOS than on aerobic glycolysis, which generates ATP using amino acids, fatty acids, and glucose as energy substrates. Because of this metabolic plasticity, LSCs survive even in hypoxic or nutrient-poor environments within the BM niche. Recently increasing studies have revealed that LSCs can evade immune surveillance by altering immune compartments and upregulating immune checkpoint molecules, such as PD-L1 (Programmed Death-Ligand 1) and CD47 [57,58,59].

Accordingly, the improvement in our understanding of LSC biology, gene expression profiles, and phenotypes resulted in novel therapeutic options aiming to eradicate LSCs within the BM.

## 6. LSCs in Other Hematological Malignancies

The role of LSCs in other hematological malignancies has also been studied by several research groups, albeit not as extensively as in AML. Herein we discuss the examples of chronic myeloid leukemia (CML), acute lymphoblastic leukemia (ALL) and multiple myeloma (MM).

In CML, LSCs are considered responsible for both disease initiation and resistance to therapy with tyrosine kinase inhibitors (TKIs) and for the evasion and progression of the disease. This resistance is primarily attributed to the quiescent nature of LSCs, which protects them from TKI therapy targeting proliferating cells, and their ability to rely on BCR::ABL1-independent survival mechanisms. These capabilities rely not only on general characteristics of LSCs, such as extrinsic support from the niche microenvironment or aberrant activation of signaling pathways like Wnt/β-catenin, Hedgehog and Notch, but also on disease-specific mechanisms [60]. The chimeric *BCR::ABL1* gene and its product are believed to disrupt several cellular processes, including cell signaling, autophagy, metabolism, and apoptosis, thus resulting in LSC survival and self-renewal. For instance, BCR::ABL1 chimeric protein can promote the expression of anti-apoptotic proteins of the BCL2 family or increase aerobic glycolysis of LSCs by activating the PI3K/AKT signaling pathway, hence supporting LSCs survival and stemness [60,61,62]. Additionally, LSCs in CML have developed several mechanisms to evade TKI therapy, some of which involve deregulated miRNA expression. Indeed, increased levels of miR-126 and miR-29a or the downregulation of the tumor suppressor miR-142 are believed to cause or enhance TKI resistance [63,64,65]. Finally, LSCs have been investigated as a potential therapeutic target in CML with several agents apart from TKIs. Indeed, pharmacological suppression of autophagy, targeting of LSC surface markers, and inhibition of signaling pathways have been proposed as potential ways to eliminate LSCs in CML and, thus, overcome first-line treatment resistance [60].

ALL generally shows greater heterogeneity and a less strict hierarchical organization compared to AML [10]. Actually, there is still uncertainty whether ALL arises from the malignant transformation of normal HSCs or from more mature lymphoid committed progenitors that have regained their stem cell properties [66]. This is because it was originally believed that proliferation and engraftment to murine models was a capacity restricted to CD34^+^/CD19^−^ immature cells, but it was later shown that stem cell properties can be found in B-ALL leukemic blasts at different maturation stages (CD19^−^ or CD19^+^) [67,68]. Even among different genetic subtypes of B-ALL, there seems to be high heterogeneity regarding the maturation stage of the cell of origin. For example, it has been demonstrated that ALL with t(12;21) as well as P190 BCR::ABL1 ALL originate from a committed B-cell progenitor, while p210 BCR::ABL1 ALL arises from the pool of less mature multipotent HSCs [69]. Less evidence is available for T-ALL, where it was shown that LICs are mainly detected in the CD7^+^/CD1a^−^ subset [70]. In any case, further research about LSCs in ALL is warranted to assess their role in disease relapse, treatment resistance, or even as a therapeutic target, similarly to AML.

In MM, the characterization of cancer stem cells (CSCs) has been rather challenging. Theoretically, multiple myeloma stem cells (MMSCs) are described as cells with the capacity to self-renew and differentiate into the neoplastic myeloma plasma cell lineages, also exhibiting drug resistance [71]. However, data regarding the laboratory identification and phenotypical characterization of these MMSCs have been conflicting. About two decades ago, circulating B-lymphocytes, which are clonally related to neoplastic plasma cells (clonotypic B-cells) were identified in patients with MM and it was suggested that they represent CSCs, since they are able to self-renew and initiate MM in murine models, although they comprise a committed cell population without the multipotent differentiation capacity that typically characterizes stem cells. Although initially identified as CD19^+^CD138^−^ (pre-PC) B-cells, later evidence indicated that CD138^+^ (plasmablasts/PC) also maintain tumor-initiating capacity [71,72,73]. Several other markers, such as CD123, aldehyde dehydrogenase, and TRIMM44 have been investigated as possible identifiers of MMSCs, without consensus on a conclusive specific phenotype, suggesting that the MMSC population is phenotypically variable and heterogenous, maybe depending on the genomic events that led to tumorigenesis [74]. Interestingly, mesenchymal stem cells (MSCs), which generate the stromal cellular compartment (osteoblasts, osteoclasts, and endothelial cells) of the BMM, have been reported to show differences in patients with MM compared to healthy individuals, such as lower proliferative capacity and abnormal cytokine expression. These MSCs in the case of MM may form a favorable microenvironment for neoplastic cells, which promotes myeloma development and progression, and are, hence, suggested as therapeutic targets but also as potential carriers of anti-tumor drugs against the actual myeloma plasma cells, due to their close cross-talk [75].

## 7. Prognostic and Predictive Implications of LSCs in AML

Soon after the discovery of the pathophysiologic role of LSCs in terms of AML treatment resistance and relapse, efforts have been made to investigate the potential prognostic and predictive utility of these cells in clinical practice.

For over a decade, it has been known that among patients with CD34^+^ AML treated with intensive chemotherapy, the burden of CD34^+^CD38^−^ LSCs at diagnosis acts as an independent prognostic factor in terms of relapse-free survival (RFS) and OS [37,76]. LSCs also showed prognostic capacity during the follow-up period, since patients in CR after one or two treatment cycles with a higher percentage of neoplastic CD34^+^CD38^−^ LSCs had shorter survival compared to those with a lower LSC percentage. Furthermore, even among MRD-negative patients, those with higher LSCs had worse outcomes compared to patients with lower LSCs, implying that MRD status and LSC burden possibly have complementary prognostic value in AML [76,77]. Similarly, it was later confirmed that patients in morphologic CR have a dismal prognosis if they remain both MRD+ and LSC+, and they should be treated as high-risk patients, regardless of their initial risk assessment according to traditional criteria [37]. However, in patients older than 60 years treated with hypomethylating agents (HMAs), the percentage of LSCs (defined as CD34+CD38− CD123^+^) at diagnosis appears not to impact prognosis, although relevant data among those treated with HMAs plus venetoclax, the current standard of care for unfit patients, are lacking [41].

Beyond quantifying LSC burden, researchers have investigated the clinical impact of specific immunophenotypic markers expressed on these cells. Clarifying the level of prognosis, current evidence indicates that high expression of LSC-associated antigens is consistently correlated with adverse clinical outcomes. For instance, Darwish et al. reported that the overexpression of LSC markers TIM-3, BMI-1, and CLL-1 in patients with AML correlates with shorter survival, albeit with the limitation that these markers are not exclusively expressed by LSCs [44]. Accordingly, patients with AML treated with intensive protocols were found to have shorter 3-year OS (18.2%) if they express at least 2 of the LSC markers CD25, CD96, and CD123, compared to patients with single or no expression of these markers (3-year OS 65%), and multivariate analysis confirmed the independent negative prognostic effect of multiple LSC marker expression [52].

Additionally, patients with high expression levels of TIM3 on CD34^+^CD38^−^ LSCs at diagnosis are expected to have lower response rates to induction chemotherapy and worse OS, compared to those with lower levels, pointing to TIM3+ LSCs as a potential prognostic and predictive biomarker [46]. Recently Huang et al. developed a risk prognostic model for patients with AML based on gene expression profiling in samples with high or low TIM3 expression, using weighted gene co-expression network analysis [47].

In routine practice, LSC-oriented MFC is most informative when measured at diagnosis (to define the aberrant stem-like phenotype) and at response timepoints (post-induction/CR assessment and pre-transplant) to identify the persistence of an aberrant CD34^+^CD38^−^ compartment. Persistently detectable LSC-enriched populations, especially when discordant with bulk MRD, should prompt closer monitoring and may support treatment intensification or trial enrollment in otherwise borderline-risk scenarios.

## 8. Therapeutic Targeting of LSCs in AML

**Figure 2:**
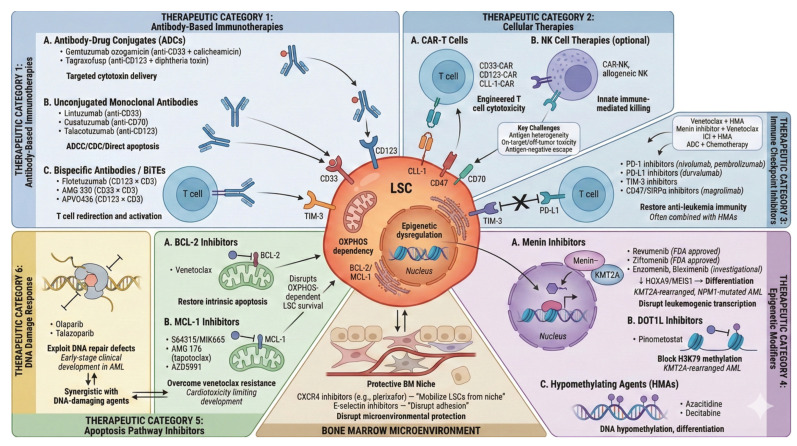
Current and emerging therapeutic strategies targeting leukemic stem cells in AML. LSCs express multiple surface antigens (CD33, CD123, CLL-1, CD47, CD70, TIM-3) that serve as targets for immunotherapeutic approaches including antibody-drug conjugates (ADCs) such as gemtuzumab ozogamicin, tagraxofusp and pivekimab sunirine, unconjugated monoclonal antibodies, bispecific T-cell engagers (flotetuzumab, AMG 330), and CAR-T cell therapies. Immune checkpoint inhibitors targeting PD-1/PD-L1 and CD47 aim to restore anti-leukemia immunity. Epigenetic therapies include menin inhibitors (revumenib, ziftomenib) that disrupt HOXA/MEIS1-driven transcriptional programs in KMT2A-rearranged and NPM1-mutated AML, and DOT1L inhibitors (pinometostat). Apoptosis pathway inhibitors target the BCL-2 (venetoclax) and MCL-1 dependencies characteristic of LSCs. PARP inhibitors exploit DNA repair vulnerabilities. Niche-targeting strategies (CXCR4 inhibitors, E-selectin inhibitors) aim to mobilize LSCs from protective bone marrow microenvironment niches. Key translational barriers include antigen overlap with normal progenitors, antigen heterogeneity, immunosuppressive microenvironment, and LSC plasticity. (Created with Gemini).

Therapeutic strategies targeting LSCs in AML are of particular interest because LSCs are implicated in chemotherapy resistance, persistence of disease, and relapse. Accordingly, LSC-directed therapy aims not only to reduce bulk blast burden but also to disrupt relapse-initiating programs and improve the depth and durability of remission. In the era of precision medicine, multiple targeted approaches are under investigation in preclinical and clinical studies, including immunotherapies, epigenetic modulators, and inhibitors of key survival and DNA repair pathways (Figure 2).

Table 2 summarizes the current landscape of therapeutic agents targeting LSCs and different stages of AML development. These agents encompass diverse mechanisms including epigenetic modulation, apoptosis pathway disruption, immunotherapy, and cell surface antigen targeting. While several agents have achieved FDA approval, many remain in clinical development, with ongoing efforts to address key barriers including on-target/off-tumor toxicity, antigen heterogeneity, and resistance mechanisms.

However, the successful application of these precision therapies relies on navigating the complex clonal architecture of AML. A critical distinction for effective eradication lies between targeting initiating (pre-leukemic) versus secondary (driver) mutations. Initiating mutations, which predominantly involve epigenetic regulators, establish a stem cell state with enhanced self-renewal and long-term persistence and may survive conventional chemotherapy, contributing to measurable residual disease and late relapse. In contrast, secondary mutations drive leukemic proliferation and differentiation arrest and therefore represent effective targets for rapid cytoreduction. However, therapies exclusively targeting secondary mutations frequently fail to eradicate pre-leukemic and LSC reservoirs, limiting their impact on long-term disease control. These findings indicate that durable remission in AML may require therapeutic strategies that integrate cytoreductive approaches with agents capable of disrupting stemness-associated epigenetic programs in disease-initiating compartments.

### 8.1. Immunotherapies

Immunotherapeutic strategies in AML aim to eliminate therapy-resistant compartments, including LSC-enriched populations, by engaging immune effector mechanisms against leukemia-associated surface antigens such as CD33, CD123, CD47, CD70, and TIM-3 [48,51]. Current approaches include antibody–drug conjugates (ADCs), unconjugated monoclonal antibodies, bispecific T-cell–engaging antibodies (BiTEs), immune checkpoint inhibitors (ICIs), and cellular therapies. A central translational constraint is that many AML-relevant antigens are also expressed on normal myeloid progenitors, narrowing the therapeutic window and necessitating careful target selection and toxicity monitoring.

#### 8.1.1. Antibody–Drug Conjugates (ADCs)

ADCs deliver cytotoxic payloads to antigen-expressing leukemic cells. Examples include gemtuzumab ozogamicin (GO), a humanized anti-CD33 monoclonal antibody (mAb) conjugated with calicheamicin and tagraxofusp which targets CD123 and is fused to diphtheria toxin [42,48]. Although these agents can induce meaningful responses, their capacity to eradicate LSC may be limited by antigen heterogeneity and by dose-limiting myelosuppression due to overlap with normal progenitor expression.

#### 8.1.2. Unconjugated Monoclonal Antibodies

Unconjugated mAbs bind leukemia-associated antigens and mediate antibody-dependent cellular/complement-dependent cytotoxicity or induce apoptosis [48]. Agents under clinical evaluation include lintuzumab (humanized IgG1 anti-CD33), cusatuzumab (anti-CD70) and talacotuzumab (second-generation anti-CD123) administered either as monotherapy or in combination regimens [51].

#### 8.1.3. Bispecific Antibodies/BiTEs

Bispecific T-cell engagers (BiTEs) recruit T cells via CD3 and redirect them to leukemic targets such as CD33 or CD123 [78]. Flotetuzumab (CD123/CD3) has shown activity in early-phase trials in adults with relapsed/refractory (R/R) AML, while other investigational bispecifics include AMG 330 (CD33/CD3) and APVO436 (CD123/CD3) agent [79]. Beyond antigen expression, the depth and durability of response may be constrained by baseline T-cell dysfunction, an immunosuppressive marrow microenvironment, and treatment-related cytopenias.

#### 8.1.4. Immune Checkpoint Inhibitors (ICIs)

ICIs targeting TIM-3, programmed death-ligand 1 (PD-L1), and CTLA-4 pathways are being explored in AML, most commonly in combination strategies (e.g., with epigenetic modifiers) in R/R disease and in older/unfit AML or MDS cohorts [48,80]. Agents under investigation include nivolumab, pembrolizumab (PD-1 inhibitors), and durvalumab (PD-L1 inhibitors) [48,80].

#### 8.1.5. Cellular Therapies (CAR-T and Related Platforms)

The role of chimeric antigen receptor (CAR) T-cell therapy in AML is being investigated under several clinical trials, focusing on antigens enriched on LSCs such as CD33, CD123, and CLL-1, with encouraging results but also notable challenges related to antigen overlap, toxicity, and disease heterogeneity. Additional targets under preclinical evaluation include myeloid markers (e.g., CD38, CD13, CD64) and NK-related targets (e.g., NKG2D, CD70) [45,80]. Key barriers include antigen overlap with normal progenitors (risk of prolonged aplasia), antigen heterogeneity/escape, and logistical challenges in rapidly progressive disease.

Across immunotherapy platforms, several barriers limit clinical efficacy: antigen heterogeneity within the LSC compartment, on-target/off-tumor myelotoxicity due to shared antigen expression on normal hematopoietic progenitors, and microenvironmental protection that shields LSCs from immune-mediated killing. These factors directly impact trial design, dosing strategies, and the rationale for combination approaches.

### 8.2. Epigenetic Modifiers

While immunotherapies aim to eliminate LSCs via antigen-directed immune engagement, epigenetic therapies target the transcriptional dependencies that maintain LSC self-renewal and differentiation blockade—dependencies that are often genotype-defined in AML.

#### 8.2.1. Menin Inhibitors

Menin is a scaffold nuclear protein, encoded by the tumor suppressor gene *MEN1* that—despite its tumor suppressor context—acts in AML as a critical cofactor sustaining leukemogenic transcriptional programs through interaction with *KMT2A*-associated complexes [81]. Because *HOXA*/*MEIS1*-driven transcription is closely linked to stemness and self-renewal, menin inhibition is of particular interest as an LSC-directed strategy, aiming to disrupt relapse-initiating circuitry rather than only reducing bulk blasts [82,83]. In *KMT2A*-rearranged AML, menin binding supports maintenance of a *HOXA*/*MEIS1* program that preserves LSC properties and enforces differentiation arrest. *NPM1*-mutated AML exhibits a related *HOX*/*MEIS1*-high expression profile, providing a strong mechanistic rationale for menin targeting in this additional genotype-defined subgroup [84,85].

Mechanistically, small-molecule menin inhibitors disrupt the menin-*KMT2A* interaction, leading to the downregulation of *HOXA9*/*MEIS1*-dependent programs, induction of differentiation, and reduction in LSC-enriched compartments. Clinically, the landscape changed materially in 2024–2025, with revumenib receiving FDA approval first in *KMT2A*-rearranged R/R acute leukemia (including pediatric patients) [86,87] and later for R/R AML with a susceptible *NPM1* mutation with no satisfactory alternatives, followed by FDA approval of ziftomenib for adults with R/R *NPM1*-mutated AML [88]. As a class, menin inhibitors require attention to differentiation syndrome and electrocardiographic monitoring considerations (e.g., QTc-related precautions), which have practical implications for early recognition and management pathways.

Beyond approved agents, several additional menin inhibitors (e.g., enzomenib, bleximenib, DS-1954, BMF-219) are under clinical evaluation as monotherapy and in combinations (e.g., venetoclax-based regimens, FLT3 inhibitors, HMAs, and intensive chemotherapy). Their ultimate positioning will likely depend on durability of response, safety profile, and combinability across molecular subsets, as well as whether deeper stemness suppression translates into sustained MRD negativity and relapse prevention [89,90,91].

#### 8.2.2. DOT1L Inhibitors

The histone lysine methyltransferase DOT1L (Disruptor of Telomeric Silencing 1-Like) is an epigenetic regulator implicated in AML with *KMT2A* rearrangements and other malignancies. Pinometostat is a first-in-class, selective, intravenous DOT1L inhibitor that demonstrated activity in early-phase clinical trials; however, responses as monotherapy have been limited, supporting ongoing evaluation of combination strategies [92,93].

### 8.3. Targeting Apoptosis Pathways

The intrinsic (mitochondrial) pathway of apoptosis is regulated by the BCL-2 protein family, which includes pro-apoptotic (Bax, Bak, Bim, Bid) and anti-apoptotic proteins (BCL-2, MCL-1, BCL-W, BCL-XL) [94]. In AML, LSCs frequently exhibit heightened dependence on anti-apoptotic programs, particularly BCL-2 and MCL-1, thereby contributing to chemoresistance and persistence of MRD.

Venetoclax, a BH3 mimetic, inhibits BCL-2 and can target LSC compartments that depend on BCL-2 and OXPHOS for survival [94,95]. However, despite the promising efficacy of venetoclax-based combination regimens in AML, resistance mechanisms—most commonly the upregulation of other anti-apoptotic proteins like MCL-1 or BCL-XL—limit the durability of responses [96]. This has driven interest in the development of MCL-1 inhibitors (e.g., S64315/MIK665, AMG 176 [tapotoclax], AZD5991), which have demonstrated preclinical activity against blasts and LSCs, although cardiotoxicity has emerged as a major limitation in clinical development [94].

### 8.4. DNA Damage Response Targeting: PARP Inhibitors

Poly (ADP-ribose) polymerase (PARP) enzymes, particularly PARP1 and PARP2, catalyze the addition of polymerized ADP-ribose onto substrates, thereby modifying their function or stability and playing a central role in DNA damage repair. PARP inhibitors such as olaparib or talazoparib exploit compromised DNA repair mechanisms in cancer cells and demonstrate synergistic activity when combined with DNA-damaging agents, including anthracyclines and topoisomerase inhibitors. In AML, however, PARP inhibitors remain in early-stage clinical development, and definitive efficacy data are not yet available [95].

## 9. Why Promising Targets Fail in AML

Despite compelling preclinical rationale and encouraging early-phase signals, many LSC-directed targets and immunotherapy platforms have not yielded durable clinical benefit in AML. These failures typically stem from the convergence of biological, microenvironmental, and clinical-trial constraints that are particularly pronounced in myeloid malignancies.

- Absence of an “ideal” AML-specific antigen: on-target/off-tumor toxicity. The most significant barrier for antibody-based and cellular therapies in AML is the lack of a surface antigen uniformly expressed on blasts and LSCs but absent from normal hematopoietic stem and progenitor cells (HSPCs). Commonly targeted antigens, CD33, CD123, and CLL-1, are also expressed on normal myeloid progenitors, creating inherent risk of prolonged myeloablation, severe cytopenias, and infectious complications when potent targeting is achieved. This shared-antigen problem represents the central limitation for CAR-based approaches in AML and explains the tight coupling between efficacy and toxicity in this disease [97,98].
- Antigen heterogeneity and antigen-negative escape: AML is biologically heterogeneous both across patients and within individual patients, characterized by dynamic subclonal evolution and variable antigen density. Consequently, single-antigen targeting strategies are therefore vulnerable to antigen-low subpopulations and antigen-negative relapse, particularly under therapeutic selective pressure. This limitation is well recognized in the CAR-T AML literature and has been specifically documented in the context of CD123-directed immunotherapies [43].
- LSC plasticity and clonal evolution under therapy: A primary reason that promising therapeutic targets fail to deliver durable responses is that the LSC state is not static. LSCs can alter their phenotype and transcriptional programs in response to treatment, and relapse frequently reflects selection of resistant subclones rather than regrowth of the original dominant population. Current LSC-focused research highlights that stemness traits are shaped by both global and subtype-specific features, and that this heterogeneity fundamentally limits the effectiveness of single-pathway or single-marker targeting strategies [99].
- An immunosuppressive BMM: The BMM in AML undermines immune effector function through multiple mechanisms, including dysfunctional antigen presentation, suppressive myeloid populations, inhibitory cytokines, and metabolic constraints. Baseline T-cell dysfunction and the immunosuppressive tumor microenvironment are increasingly recognized as key factors contributing to the limited and variable clinical activity observed with AML immunotherapies, including CD123-directed approaches [43,100].
- High-risk disease biology can overwhelm single-agent promise (example: *TP53*-mutated AML): Clinical failures are often most evident in genomically adverse disease, where aggressive biology and profound treatment resistance limit the ability of any single mechanism to meaningfully improve survival. The phase 3 ENHANCE-2 trial of magrolimab plus azacitidine in previously untreated *TP53*-mutated AML provides a recent example: despite encouraging signals from non-randomized studies, the combination failed to improve OS compared with physician’s choice [50].
- Trial-design and implementation constraints in AML: Even effective modalities face significant practical barriers in AML: patients may deteriorate rapidly, have active infections, or carry substantial prior-treatment burdens that compromise immune function and organ reserve. For cellular therapies, logistical challenges, including manufacturing time, bridging therapy requirements, and toxicity management, combined with the need to avoid prolonged aplasia, can limit feasibility and confound outcome interpretation. These factors represent core obstacles to the broad applicability of CAR-based approaches in AML.

## 10. Discussion

AML remains a major clinical challenge, in large part because LSCs can persist despite therapy and re-initiate disease after apparent remission [101]. LSCs are rare but highly resilient, characterized by phenotypic and functional heterogeneity, quiescence, metabolic plasticity, and multiple mechanisms of treatment resistance—features that collectively contribute to chemoresistance, MRD persistence, and ultimately relapse [102]. Although therapeutic options have expanded substantially with the introduction of targeted agents and novel combinations, outcomes remain suboptimal, particularly in older or medically unfit patients. These observations underscore an unmet need for a more integrated understanding of AML biology that links LSC properties to clinically actionable biomarkers and rational therapeutic strategies.

The most important challenge regarding the clinical utility of LSCs is the phenotypic resemblance between LSCs and normal HSCs, which complicates their accurate distinction and identification [56]. However, MFC has emerged as a vital tool in routine practice, enabling the identification of LSCs through aberrant immunophenotypes, including LSC-specific markers such as CD34^+^CD38^−^, CD123, TIM-3, CLL-1, and CD96 [36]. Incorporating these markers into MFC-based MRD assessments has significantly enhanced risk stratification and the early detection of relapse [103].

**Figure 3:**
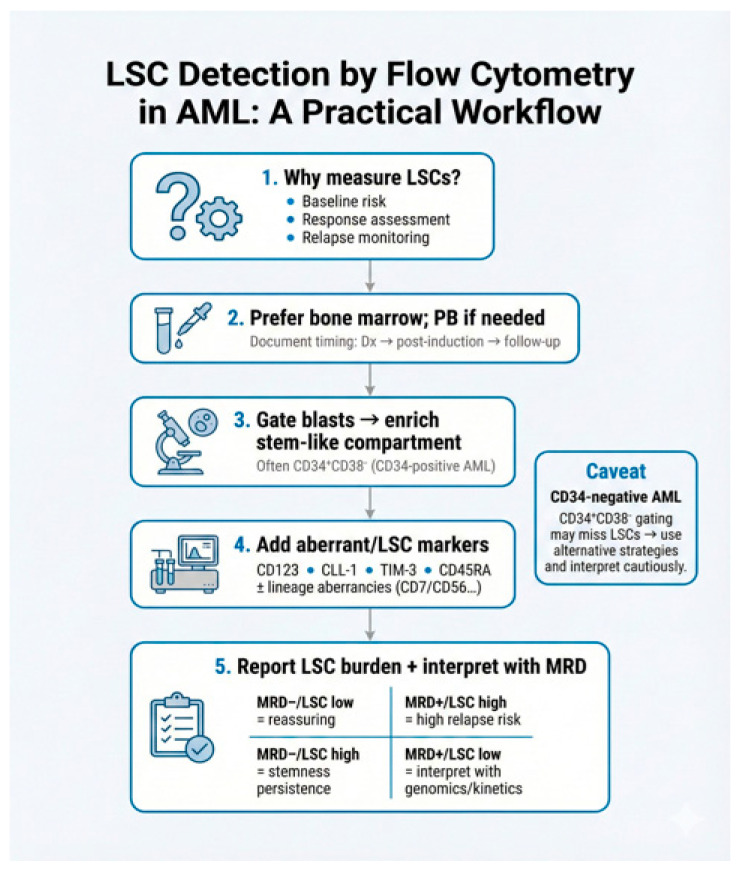
Practical workflow for MFC-based LSC detection and its integration with MRD in AML. The workflow illustrates a stepwise approach to LSC assessment, beginning with clinical rationale (step 1), sample selection with preference for bone marrow (step 2), gating strategy to enrich for the stem-like CD34^+^CD38^−^ compartment (step 3), and incorporation of aberrant LSC-associated markers (step 4). Step 5 integrates LSC burden with measurable residual disease (MRD) status for clinical interpretation: MRD^−^/LSC low indicates favorable prognosis; MRD^+^/LSC high signals high relapse risk; discordant results (MRD^−^/LSC high or MRD^+^/LSC low) require integration with genomic and kinetic data. The caveat box highlights that CD34-negative AML cases may harbor LSCs outside the conventional CD34^+^CD38^−^ gate, necessitating alternative detection strategies. Abbreviations: AML, acute myeloid leukemia; CLL-1, C-type lectin-like molecule-1; Dx, diagnosis; LSC, leukemic stem cell; MFC, multiparameter flow cytometry; MRD, measurable residual disease; PB, peripheral blood; TIM-3, T-cell immunoglobulin and mucin domain-3.Abbreviations: AML, acute myeloid leukemia; BM, bone marrow; HSC, hematopoietic stem cell; LSC, leukemic stem cell; MFC, multiparametric flow cytometry; MRD, measurable residual disease; NGS, next-generation sequencing; PB, peripheral blood; PCR, polymerase chain reaction.

Our review highlights three critical clinical scenarios that arise from this dual-monitoring approach, alongside a fourth distinct pattern (Figure 3).

The Discordant “Hidden Risk” (MRD^−^/LSC^+^): Patients who achieve morphological and MRD-negative status but retain a detectable LSC compartment face significantly shorter survival [36,77]. Canali et al. identified this group as comprising 25.8% of their cohort, with a 3-year OS of only 52% compared to 88% in double-negative patients [77]. In these cases, LSCs act as a “reservoir” for future clones, implying that MRD negativity alone may be insufficient to declare a durable remission [55,104].

The Ultra-High Risk (MRD^+^/LSC^+^): The persistence of both bulk blasts and a stem-like compartment indicates profound treatment resistance. Zeijlemaker et al. demonstrated that these patients have a hazard ratio of 3.62 for OS and 5.89 for cumulative incidence of relapse, with nearly 100% treatment failure probability [36]. These patients should be considered for immediate treatment intensification or stem cell transplantation (SCT), regardless of their initial ELN risk category.

The Persistent Bulk Risk (MRD^+^/LSC low): This category represents patients with persistent MRD despite a low burden of phenotypically detectable LSCs. Clinically, the significance of this group lies in the fact that measurable disease persistence itself is a strong driver of relapse. Even if the specific LSC fraction appears low, the presence of residual leukemic bulk indicates that induction therapy failed to achieve deep remission, placing these patients in a high-risk category driven by the active disease burden [36].

The Low-Risk Transition (MRD^−^/LSC^−^): This status represents the optimal therapeutic goal: a “deep” remission where the relapse-initiating programs have been effectively eradicated [36,77].

Biologically, LSCs exhibit several survival strategies that render them refractory to standard chemotherapy and responsible for disease relapse [57,59]. Their quiescent nature allows them to evade cell cycle-specific agents like cytarabine and anthracyclines [105,106]. Elevated efflux pump activity, particularly through ATP-binding cassette (ABC) transporters such as ABCG2 and P-glycoprotein (MDR1), reduces intracellular drug accumulation. Overexpression of anti-apoptotic proteins like BCL-2, MCL-1, and survivin further fortifies LSCs against apoptosis [102]. Additionally, enhanced DNA repair mechanisms [57,59] and a reliance on OXPHOS over glycolysis endow LSCs with superior metabolic resilience [13,14]. Their localization within protective bone marrow niches, which offer anti-apoptotic and immune-privileged signals [30,34], and the expression of immune evasion molecules such as PD-L1 and CD47 [57,59], further contribute to their long-term survival and escape from both therapy and immune surveillance.

These unique features of LSCs have stimulated the development of novel targeted therapies aimed at their selective eradication while allowing normal HSCs survival. Epigenetic regulators have emerged as critical targets: menin inhibitors, such as revumenib and ziftomenib, disrupt the oncogenic KMT2A-fusion/menin interaction, downregulating HOXA9 and MEIS1 and impairing LSC self-renewal in *KMT2A*-rearranged and *NPM1*-mutated AML [83,84,85], while DOT1L inhibitors like pinometostat block H3K79 methylation, further suppressing leukemogenic gene expression in *KMT2A*-rearranged AML [92,93]. Immunotherapeutic approaches offer complementary strategies for LSC elimination. Unconjugated monoclonal antibodies targeting antigens such as CD33 (lintuzumab), CD123 (talacotuzumab), and CLL-1 have shown activity in early-phase trials [48,51]. ADCs, such as gemtuzumab ozogamicin (anti-CD33) and pivekimab sunirine (IMGN632; anti-CD123), deliver cytotoxic agents directly to leukemic cells, enhancing efficacy while limiting systemic toxicity [48]. Bispecific antibodies, such as flotetuzumab (CD123/CD3), recruit T cells to eliminate LSCs, representing a novel immunotherapeutic approach in AML treatments [42,78]. Immune checkpoint inhibitors targeting PD-1/PD-L1 and CD47 are under investigation to reverse LSC-mediated immune escape and reinvigorate anti-leukemic immunity [48,80]. CAR T -cell therapies directed against LSC-associated antigens such as CD33, CD123, and CLL-1 are also being explored, though challenges remain regarding on-target, off-tumor toxicity and antigen escape [43,45,97,98]. Further refinement of CAR-T cell constructs and targeting strategies is essential for their safe and effective implementation in AML. Targeting metabolic and survival mechanisms of LSC has increasingly become a focus of research. BCL-2 inhibitors such as venetoclax disrupt the intrinsic apoptotic pathway, preferentially affecting LSCs due to their dependency on BCL-2 proteins [94,95]. However, resistance due to the upregulation of MCL-1 is a frequent occurrence, prompting the development of selective MCL-1 inhibitors (e.g., S64315, AZD5991) to overcome resistance and act synergistically with venetoclax [94,96]. Finally, PARP inhibitors are being evaluated for their potential to exploit DNA repair vulnerabilities in LSCs, particularly in cases with defective homologous recombination repair [95].

Despite these advances, several unmet needs persist. LSCs continue to evade current treatments through their adaptability, microenvironmental protection, and phenotypic plasticity. There is a critical need for biomarkers to dynamically track LSC burden, guide therapy, and predict relapse early. Furthermore, as most novel agents remain in early-phase trials, their long-term efficacy and safety remain to be fully established. Integrating multi-omic approaches, real-time single-cell analyses, and functional assays, probably with the use of artificial intelligence, will be key to unveiling the evolving LSC landscape and informing precision medicine strategies.

## 11. Conclusions

In conclusion, while the identification and targeting of LSCs have progressed significantly, achieving their complete eradication to minimize the risk of clinical relapse remains the “Holy Grail” of AML therapy. The integration of standardized LSC-enriched MFC panels for dynamic risk stratification with metabolic targeting, immunotherapy, and epigenetic modulation holds the greatest promise for transforming treatment outcomes. By bridging bench biology and clinical decision-making through continued translational research and innovative trial designs, we can shift from simply treating the disease to preventing relapse at its source.

## Figures and Tables

**Table 1:** Summary of reported immunophenotypic markers distinguishing LSCs from HSCs in AML based on the current literature. +: expressed, ++: highly expressed, −: negative, dim: low expressed, +/−: variable expression, LSCs: leukemic stem cells, HSCs: hematopoietic stem cells, BM: bone marrow, CAR-T cell: chimeric antigen receptor T-cell. Abbreviations: ADC, antibody-drug conjugate; BiTE, bispecific T-cell engager; BM, bone marrow; CAR-T, chimeric antigen receptor T-cell; HSC, hematopoietic stem cell; LSC, leukemic stem cell; mAb, monoclonal antibody; MRD, measurable residual disease; NK, natural killer.

Marker Category	Marker	Expression on LSC	Expression on HSC	Distinction Role and Clinical Relevance	Therapeutic Relevance	References
Backbone and Stemness	CD34	+ (variable)	++	Present in both LSCs and HSCs; requires additional markers for specificity; starting marker for gating	—	[4,5,29,30,31,32,33]
	CD38	−	+	LSCs are CD34^+^CD38^−^, while HSCs express CD38	CD38-directed therapies (e.g., daratumumab) under investigation	[4,5,29,30,31,32,33]
	CD90 (Thy-1)	−	+	Critical negative marker; distinguishes LSCs from normal HSCs (CD90+); essential for LSC-MRD detection	—	[31,32,33,38]
	CD45RA	+/−	−	Key aberrant marker; helps distinguish LSCs from normal HSCs; enriched in certain AML subtypes	—	[30,31,37]
Surface Antigens	CD123 (IL-3Rα)	++	dim	Overexpressed in LSCs compared to HSCs; key diagnostic and prognostic marker	Target of tagraxofusp (ADC), talacotuzumab (mAb), flotetuzumab (BiTE), CD123-CAR-T	[30,31,37,41,42,43]
	CLL-1 (CLEC12A)	+	−	Absent on normal HSCs; high specificity for LSCs	Ideal target for CAR-T cell therapy; ADCs in development	[30,31,37,44,45]
	TIM-3 (CD366)	++	−	Enriched in LSCs; associated with poor prognosis and therapy resistance	Target of sabatolimab (anti-TIM-3 mAb); Phase II/III trials	[6,30,44,46,47,48]
	CD33	+	+	Expressed on myeloid lineage; variable expression on LSCs	Target of gemtuzumab ozogamicin (ADC), lintuzumab (mAb), AMG 330 (BiTE), CD33-CAR-T	[30,31,45,48]
	CD44	++	+	Crucial for LSC interaction with BM niche; adhesion molecule	Anti-CD44 antibodies in preclinical development	[30,31]
Immune Evasion	CD200	++	+	“Don’t kill me” signal; suppresses T-cell & NK-cell activity; associated with poor prognosis	Anti-CD200 antibodies in early development	[32,33,34]
	CD47	++	+	“Don’t eat me” signal; overexpressed in LSCs; allows evasion of phagocytosis; high expression linked to venetoclax resistance	Target of magrolimab (anti-CD47 mAb); Phase III trials (ENHANCE-2)	[10,40,49,50]
	CD70	++	−	Supports LSC survival via CD70-CD27 immune modulation axis	Target of cusatuzumab (anti-CD70 mAb); Phase I/II trials	[51]
Other Markers	CD96	+/−	dim	Higher in LSCs; implicated in adhesion and survival	Potential target; preclinical investigation	[32,52]
	CD117 (c-KIT)	+/−	+	Expressed in some LSC populations; receptor tyrosine kinase	—	[32,33,34]
	CD7	+	−	Aberrant expression in AML; linked to poor prognosis and therapy resistance	CD7-CAR-T in development (mainly for T-ALL)	[30,31]
	CD9	++	+/−	Associated with drug resistance and stemness in LSCs	—	[32,33,34]
	HLA-DR	+/−	+	Associated with poor prognosis in HLA-DR negative AML subtypes	—	[32,33,34]
	CD99	++	dim	LSC adhesion, migration, and potential immune evasion	Anti-CD99 antibodies in preclinical studies	[32,33,34]
	IL1RAP	++	−	Interleukin-1 receptor accessory protein; LSC-specific marker	Potential therapeutic target; preclinical	[32,33,34]
	CD244	+	+/−	Immune evasion and survival of LSCs	Potential therapeutic target	[32,33,34]
	CD56	++	+/−	Promotes leukemogenesis; associated with extramedullary disease	Potential therapeutic target	[30,31,32,33,34]

**Table 2:** Summary of therapeutic agents targeting LSCs and different stages of AML development.

Therapeutic Class	Agent(s)	Target/Mechanism	AML Stage/Population	Development Status
Menin inhibitors	Revumenib	Menin-KMT2A interaction; downregulates HOXA9/MEIS1	LSCs in KMT2A-r and NPM1-mutated AML	FDA approved for R/R KMT2A-r and NPM1-mutated AML
	Ziftomenib	Menin-KMT2A interaction	LSCs in NPM1-mutated AML	FDA approved for R/R NPM1-mutated AML
	Enzomenib, Bleximenib, DS-1954, BMF-219	Menin-KMT2A interaction	LSCs in KMT2A-r/NPM1-mutated AML	Phase I/II clinical trials
BCL-2 inhibitors	Venetoclax	BCL-2; disrupts mitochondrial apoptosis	LSCs dependent on OXPHOS and BCL-2	FDA approved in combination with HMA or LDAC for newly diagnosed AML in unfit patients
MCL-1 inhibitors	S64315/MIK665, AMG 176, AZD5991	MCL-1; overcomes venetoclax resistance	LSCs with MCL-1 dependency or venetoclax resistance	Phase I/II; limited by cardiotoxicity
Antibody-drug conjugates	Gemtuzumab ozogamicin	CD33; delivers calicheamicin	CD33+ blasts and LSCs (variable)	FDA approved for CD33+ AML
	Tagraxofusp	CD123; delivers diphtheria toxin	CD123^+^ LSCs	FDA approved for BPDCN; Phase I/II in AML
	Pivekimab sunirine (IMGN632)	CD123; delivers DNA-alkylating payload	CD123^+^ LSCs	Phase II clinical trials
Bispecific T-cell engagers	Flotetuzumab	CD123 × CD3; T-cell redirection	CD123^+^ blasts and LSCs	Phase I/II; promising in R/R AML
	AMG 330	CD33 × CD3; T-cell redirection	CD33^+^ blasts	Phase I clinical trials
Immune checkpoint inhibitors	Nivolumab, Pembrolizumab	PD-1; restores T-cell function	Reversing LSC-mediated immune evasion	Phase I/II; often combined with HMAs
	Sabatolimab	TIM-3; enhances anti-leukemia immunity	TIM-3^+^ LSCs	Phase II/III clinical trials
	Magrolimab	CD47; blocks “don’t eat me” signal	CD47^+^ LSCs and blasts	Phase III (ENHANCE-2) did not meet primary endpoint in TP53-mutated AML
CAR-T cell therapies	CD33-CAR-T, CD123-CAR-T, CLL-1-CAR-T	Respective surface antigens; T-cell cytotoxicity	Antigen+ LSCs and blasts	Phase I/II; limited by on-target/off-tumor toxicity and antigen escape
DOT1L inhibitors	Pinometostat	DOT1L; blocks H3K79 methylation	LSCs in KMT2A-rearranged AML	Phase I/II; limited single-agent activity; combination trials ongoing
PARP inhibitors	Olaparib, Talazoparib	PARP1/2; exploits DNA repair defects	LSCs with HR deficiency	Early-phase trials; definitive efficacy data not yet available
Unconjugated monoclonal antibodies	Lintuzumab	CD33; ADCC/CDC	CD33^+^ blasts	Phase II/III; limited single-agent efficacy
	Cusatuzumab	CD70; disrupts CD70-CD27 axis	CD70^+^ LSCs	Phase I/II in combination with HMAs
	Talacotuzumab	CD123; ADCC	CD123^+^ LSCs	Phase II/III; development discontinued
